# Knowledge, Attitude and Behaviours Related to Weight Control and Body-Image Perceptions among Chinese High School Students

**DOI:** 10.21315/mjms2019.26.5.11

**Published:** 2019-11-04

**Authors:** Chia Yin Lee, Hayati Mohd Yusof, Noor Salihah Zakaria

**Affiliations:** School of Food Science and Technology, Universiti Malaysia Terengganu, Kuala Nerus, Terengganu, Malaysia

**Keywords:** body image, adolescents, weight control, Chinese

## Abstract

**Background:**

Body-image perception is one of the determinants in weight management, especially among adolescents. This study aimed to assess weight-control knowledge, attitude and behaviours along with body-image perceptions among Chinese high school students in order to compare the weight-control behaviours with those perceptions.

**Methods:**

A cross-sectional study was conducted among 277 Chinese students in Form 1 and Form 2 in Pulau Pinang, Malaysia using convenience sampling. The following outcomes were evaluated: weight-control knowledge, attitude and behaviours (weight-related knowledge and attitude questionnaire; weight control strategies scale) and body-image perceptions (figure rating scale).

**Results:**

Both genders were found to have high weight-control knowledge, with female adolescents scoring significantly higher than male adolescents (*P* = 0.010). However, only half of the adolescents (50.9%) perceived that obesity is bad for health. Although only 44.4% of adolescents were dissatisfied with their current weight status, 62.8% intended to change their present weight status. Male adolescents significantly engaged more in physical activity (PA) (*P* = 0.035) and self-monitoring (SM) (*P* = 0.014) compared to their female counterparts. Furthermore, male adolescents chose their current body size as their ideal body image, but female adolescents preferred a slimmer ideal figure. The percentage of male and female adolescents who desired a smaller body figure was 39.6% and 54.5%, respectively. Lastly, there was no significant difference between weight-control behaviours and adolescents’ body-image perceptions.

**Conclusion:**

Female Chinese adolescents had higher weight-control knowledge and preferred a slimmer body size, yet males were more likely to engage in PA and SM behaviours. Essentially, imprecise attitude towards obesity among half of the Chinese high school students is of particular concern.

## Introduction

The childhood obesity epidemic is worsening worldwide, including in Asian countries. In Malaysia, the percentage of overweight and obese children as well as adolescents between ages 5 and 19 escalated from 0.3% to as high as 12.7% in 2016 (1). This chronic health issue takes a toll on the affected children and adolescents not only physically but psychologically as well.

Appropriate weight-control practices should be promoted to prevent obesity among adolescents. According to López-Guimerà et al. (2), weight-control strategies adopted by adolescents include healthy practices, such as increased consumption of vegetables and fruits, reduced intake of sugar-sweetened beverages, and increased physical activities (PAs), but some may practise unhealthy behaviours that bring more harm than good to the body, for example, dieting, skipping meals and even self-induced vomiting diet pills (3). In addition, it was reported that weight-control knowledge is positively related to weight-loss behaviour (4), which may help in reducing the prevalence of obesity among adolescents.

Body image is another factor that may lead to weight-related concerns among adolescents. Khor et al. (5) reported that 87% of adolescents in Pulau Pinang and Kedah were concerned with their body shape and that this in turn would lead to the development of individual image. Study has shown that more adolescent girls tend to select an underweight figure as their ideal body size than boys do (6). This distorted body-image perception is further reinforced by the use of social media (7, 8).

Previous studies have reported that Malaysian Chinese males and females were more dissatisfied with their body shape than different races, despite the fact that the Chinese have the lowest percentage of obesity in Malaysia, compared to Malays and Indians (9, 10). Although there have been a few recent studies conducted among Malaysian adolescents related to weight-control practices (4, 6, 11) and body-image perceptions (5, 12), there is still insufficient information on the knowledge level and attitude of adolescents regarding weight control, especially among Chinese adolescents. Moreover, there are also limited resources available that address the differences between weight-control behaviours and different body-image perceptions, particularly among Malaysian adolescents.

Studying weight-control knowledge, attitude, behaviours and body-image perceptions among Chinese adolescents may help increase the awareness about body-image issues and weight-related concerns in this cohort so that their health is not jeopardised, as adolescence is gradually being known as a key life stage facing difficulty in treating disease and improving health (13). Therefore, this research aims to assess the knowledge, attitude and behaviour related to weight control and body-image perceptions among Chinese high school students between genders as well as to compare the weight-control behaviours with adolescents’ body-image perceptions.

## Materials and Methods

### Study Design, Sampling Plan and Sample

A school-based cross-sectional study using convenience sampling was conducted at two Chinese high schools in Pulau Pinang between early July and mid-July 2018. Eligibility to participate in this study required students to be Chinese, either in Form 1 or Form 2 at the selected high schools and able to understand English. A minimum sample size was calculated and found that at least 271 respondents were required to participate in this study, with a precision error of 4% and at type 1 error of 5% (14). The expected proportion of the population was estimated based on the prevalence of body-image concerns among adolescents in Kedah and Pulau Pinang (87.0%) (5).

### Research Instrument

A self-administered questionnaire was used as the research instrument for this study. It consisted of six sections and was administered in English. Sociodemographic data and anthropometric measurements (i.e., weight and height) were self-reported by the students. The validity of self-reported weight and height among Malaysian secondary school children has been previously verified (15). Age and sex standardised body mass index (BMI) Z-scores were defined according to World Health Organization (WHO) growth standards, which classify overweight as Z-score>+1SD, obesity as Z-score>+2SD and thinness as Z-score<−2SD (16).

Weight-control knowledge was assessed using questions established by Cai et al. (17). There was a total of 10 items relative to dietary intake (8 items), exercise (1 item) and screen time (1 item). Subjects were given three responses for all questions that were ‘true’, ‘false’ or ‘don’t know’. For every correct answer, one point was given, and zero was given for an incorrect response. The total number of correctly answered questions was summed and calculated as the knowledge score, which ranged from 0 to 10. A higher score indicated a higher level of knowledge related to weight control.

Weight-control-related attitude was evaluated by asking four questions adapted from Cai et al. (17). The questions were as follows: (i) To what extent do you think obesity is bad for health? (ii) Are you satisfied with your weight status? (iii) Do you want to change your present weight status? (iv) Do you believe you can achieve an ideal weight status through effort? The responses given for the first question were ‘little’, ‘rather little’, ‘not sure’, ‘rather greatly’ or ‘greatly’, whereas ‘no’, ‘rather no’, ‘not sure’, ‘rather yes’ or ‘yes’ were given for the remaining questions. Responses of ‘rather greatly’ and ‘greatly’ for the first item indicated that the respondents expressed concerns towards obesity. Meanwhile, dissatisfaction with weight status was shown when respondents chose ‘no’ or ‘rather no’ for the second question. When ‘rather yes’ or ‘yes’ was answered for the last two questions, this indicated that respondents wished to change their current weight status and that they were confident they could achieve an ideal weight status through effort.

Weight-management behaviours were measured using the weight control strategies scale (WCSS) developed and validated by Pinto et al. (18). The questionnaire consisted of four different domains assessing how frequent subjects engage in weight management activities: (i) behavioural skills, (ii) dietary behaviours, (iii) physical activity behaviours and (iv) psychological coping skills. There was a total of 30 items; 10 items were related to dietary choices (WCSS-DC), seven items to self-monitoring strategies (WCSS-SM), six items to physical activity (WCSS-PA) and seven items to psychological coping (WCSS-PC). A five-point Likert scale was used to rate each item, with 0 = Never, 1 = Occasionally, 2 = About half of the time, 3 = Most of the time and 4 = Always. To obtain a total WCSS score ranging from 0 to 120, all item scores were added and divided by 30. Then, the item scores for each subscale were summed and divided by the number of items in that subscale. Then, the total scores obtained by the subjects were compared with the total scores of each subscale. A higher score indicated that the individual more frequently engaged in weight-management activities.

Body-image perception was evaluated using a questionnaire adapted from Stunkard et al. (19) that consisted of nine silhouette drawings. The scores for figures ranged from 1 to 9, with 1 = thinnest body size and 9 = biggest body size. Two different scales for male and female silhouettes were used to assess boys’ and girls’ body-image perceptions. Students selected the most suitable body size that defined their current body size as well as their ideal body size from a series of figures in the figure rating scale (FRS). The deviation represented the respondents’ satisfaction with their own body image, and this was calculated as the difference between the perceived current body image and ideal body image. A zero score corresponded to body satisfaction, whereas a positive value indicated the respondent’s desire to be bigger than the current size and a negative value reflected the respondent’s preference for a smaller figure (20).

### Data Collection Procedure

Before study commencement, permission was obtained from Kementerian Pendidikan Malaysia (KPM) and Jabatan Pendidikan Negeri Pulau Pinang (JPNPP). After obtaining permission from the school principals, data collection was scheduled during normal school hours under the supervision of the investigator. Eligible students who gave written consent were briefed about the study. The completed questionnaires were then collected all at once. Students were also requested to fill in any missing data after the completed questionnaires were checked.

### Data Analysis

The collected data were analysed using IBM Statistical Package for Social Sciences (SPSS) version 20.0. All descriptive statistics were reported in frequencies and percentages. The Mann-Whitney U test was employed to compare the weight-control behaviours and body-image perceptions between genders. The Kruskal-Wallis test was also used to determine if there were statistically significant differences between the three groups of body-image perceptions. A *P*-value < 0.05 was considered statistically significant.

## Results

### Sociodemographic and Anthropometric Measurement

A total of 277 students participated, comprising 55.6% male and 44.4% female. There were more Form 2 students (56.7%) than Form 1 (46.3%). The median BMI was 18.7 kg/m^2^ (IqR = 5.0). Based on WHO classification, the majority had normal weight (62.8%), 13.7% were overweight and 9% were obese. Of note, the prevalence of thinness was 9%.

### Weight-Control Knowledge, Attitude and Behaviour

Table 1 shows the number of correct answers scored by the Chinese adolescents for each weight-control-related knowledge item. Females had a higher correct rate for most questions, as compared to males, except for questions regarding exercising and milk consumption. More than half of all students answered the question regarding meat consumption incorrectly, with only 47.7% answering ‘no’ when asked whether more meat intake is better. The correct rate for this question was particularly low for males (42.2%) as compared to females (54.5%). In addition, over half of the students (males, 66.9% and females, 65.9%) thought that it is not necessary to exercise daily. Generally, these students had a high level of weight-control knowledge, considering the median scores for both genders were 8 out of a total score of 10. There was a significant difference in the weight-control knowledge between genders, as the mean rank score for females was significantly higher than that of males (152.76 versus 128.01; *P* = 0.010).

Table 2 presents the attitude of Chinese adolescents related to weight control. It is important to note that only half (50.9%) considered obesity to be bad for health. However, although only 44.4% were found to be dissatisfied with their weight status, more than half of the adolescents (62.8%) intended to change their present weight status, with a higher percentage of females (69.1%) than males (57.8%) feeling this way. In terms of achieving their ideal weight status through effort, 57.7% female and 51.3% male adolescents showed high confidence levels.

The most frequent strategies for weight-control behaviour used by both males and female students were dietary choices, while self-monitoring strategies were the least commonly practised by the students (Table 3). Male adolescents were significantly found to be more engaged in self-monitoring (*P* = 0.014) and physical activity (*P* = 0.035) than their female counterparts.

### Body-Image Perception

Only one-third (33.9%) of the Chinese adolescents were satisfied with their current body image (Table 4). A higher percentage of male adolescents (39.6%) preferred a smaller body figure, as compared to 29.2% of those who desired a bigger body figure. When assessing their ideal body-image perceptions using Stunkard’s figure rating scale, most of male and female adolescents chose silhouette 4 as their current body image (Table 5). Males selected the same figure size, indicating that there was no difference between their current and ideal body-image perceptions. However, females in general preferred silhouette 3 as their ideal body image, which was smaller in size as compared to the body size they had chosen as their current body image. However, further assessment showed that there was no significant difference between weight-control behaviours and adolescents’ body-image perceptions (Table 6).

## Discussion

This study described the weight control knowledge, attitude and behaviour in a sample of Chinese adolescents in Pulau Pinang. Despite having high level of weight control knowledge and engaging in frequent weight control behaviour, imprecise attitude towards obesity was identified among this group of adolescents. A smaller body figure was also preferred by them.

In opposition to previous findings (6, 23), this study found that the majority of the adolescents had rather high levels of weight-control knowledge. The high prevalence of body-image concerns among female adolescents may be one of the contributors to the high levels of weight-control knowledge among female adolescents. However, it is important to note that most of the male and female adolescents did not answer the question regarding meat consumption correctly. As claimed by Harris et al. (21), consumption of red meat is associated with the development of lean muscle tissue in German male adolescents. Hence, male adolescents in this study may have the impression that the more they eat meat, the better it is for their body. While more than half of the adolescents thought that it is not necessary to exercise on a daily basis, it has been proposed that personal factors, such as hot weather, lack of an exercise partner, lack of time, lack of skill to carry out exercise, laziness and unavailability of facilities and equipment, may lead to physical inactivity among adolescents (22).

In terms of weight-control attitude, only half of the adolescents considered obesity as being bad for health. This shows that the majority of the adolescents in this study did not identify obesity as a serious health condition that would increase the risk of other health problems, and this finding is rather concerning, as the prevalence of obesity among children and adolescents in Malaysia has been on the rise since 1975 and obesity during adolescence tends to persist into adulthood if no action is taken (1, 6). To tackle this problem, school authorities should comply with the policies developed by the Malaysian government by providing healthier food choices and suitable facilities as well as equipment for students to engage in physical activity (24).

Based on the results, it was observed that dissatisfaction with their weight status may not be the sole reason for the adolescents’ intention to change their weight status. O’Dea et al. (25) also reported a similar result in their study where as high as 80% of Australian girls attempted to lose weight when only 40% of them perceived themselves as ‘too fat’. Parental pressure, including comments about their children’s appearance, may cause children to be more conscious about their body image as well as body weight (26). In addition, adolescents’ self-esteem also plays a role in how they view their bodies. The higher their self-esteem level, the more they perceive their body image positively (27).

The Chinese adolescents in this present research were also more likely to make dietary choices in favour of losing weight, for example, avoiding high calorie and fried foods, limiting intake of sweetened beverages and increasing intake of fruits and vegetables daily, as compared to other types of weight-control behaviours. Nonetheless, they did not participate in weight-control behaviours frequently, given that their scores were relatively low. Such finding is in contrast with that of previous findings for adolescents in other countries that reported almost half of the adolescents engaged in weight-control behaviours (28, 29). When comparing genders, male adolescents were significantly more involved in weight-control behaviours in terms of self-monitoring and physical activity than female adolescents. Aniza et al. (22) and Wahida et al. (6) have presented similar results as well.

In agreement with previous results (5, 28, 30, 31) this study also found that female Chinese adolescents would prefer a smaller body figure as their ideal body image. Puberty accompanied by physical changes in terms of body fat distribution moves adolescent girls further away from the societal beauty standard of unrealistic thinness, while the increase in lean muscle mass that happens to boys causes them to be more satisfied with their current body image as this muscle gain helps build a larger body shape (5).

Interestingly, this research also discovered that more male Chinese adolescents would prefer a smaller ideal body image than a bigger body figure. This is not consistent with the gender norms stated in previous studies (5, 28). A study in Ireland also claimed a similar finding (31). According to Xu et al. (32), Asian culture does not hold muscularity as a cultural standard for males as Western culture does. Furthermore, Korean popular (K-pop) culture, which features thin female idols and male idols with soft masculinity, may have affected male adolescents’ perception of body size since K-pop culture is more influential to Malaysians nowadays (33, 34).

No significant difference was observed in terms of weight-control behaviours and Chinese adolescents’ body-image perception. This shows that the frequency and type of weight-control behaviours did not differ no matter how these adolescents perceived their body image. However, Chongwatpol and Gates (35) suggested that Thai adolescents who preferred a thinner body figure engaged the most in weight-control practices than those who were satisfied and desired a bigger body figure. At the same time, Symons et al. (36) claimed that those who were satisfied with their body image tended to engage more in physical activity than those who perceived themselves as too fat or too thin. These previous studies showed that different body images would influence an individual’s frequency of participating in weight-control behaviours.

There are certain limitations in this study which restrict its conclusion. Recruitment of respondents using convenience sampling and being restricted to only two locations may not be representative of all Chinese adolescents. In addition, weight-control behaviour was self-reported and, hence, were subject to recall bias. Additional objective measures should be used for future trial to provide more comprehensive data.

## Conclusion

The present study demonstrated that Chinese adolescents had a relatively high level of knowledge regarding weight control, but gender difference existed, as female adolescents had significantly higher levels of weight-control knowledge. Despite that, both genders had imprecise attitudes towards weight control, with only half of them thinking that obesity was bad for health and more of them wishing to change their present weight status whether they were dissatisfied with their weight status or not. Male adolescents were found to participate more in weight-control behaviours in terms of physical activity and self-monitoring than their female counterparts. Chinese adolescents were also concerned with their body image, as a high percentage of male and female adolescents had a negative body-image discrepancy score, which indicates that they preferred a smaller body image. However, a majority of male adolescents chose their current body image as their ideal body image rather than choosing a smaller body figure, as the female adolescents did. Still, there was no significance difference between weight-control behaviours and adolescents’ body-image perceptions.

## Figures and Tables

**Table 1:** Number and percentage of correct answer for each weight-control knowledge item for male and female

Knowledge items	Number (% of correct answer)		
	Male (*n* = 154)	Female (*n* = 123)	Total (*n* = 277)
It is harmful to watch TV or play computer for a long time. [yes]	106 (68.8)	99 (80.5)	205 (74.0)
It is not necessary to exercise every day. [no]	103 (66.9)	81 (65.9)	184 (66.4)
Fruits and vegetables should be eaten every day. [yes]	136 (88.3)	117 (95.1)	253 (91.3)
Meat contains fat and protein and the more you eat meat, the better. [no]	65 (42.2)	67 (54.5)	132 (47.7)
It is healthier to drink plain boiled water than sugar-sweetened beverages. [yes]	124 (80.5)	110 (89.4)	234 (84.5)
You don’t have to eat breakfast as long as you eat more lunch. [no]	125 (81.2)	102 (82.9)	227 (81.9)
It is harmful to eat too much fried food. [yes]	123 (79.9)	109 (88.6)	232 (83.8)
Western fast food (i.e. KFC, McDonald’s, etc) is more nutritious. [no]	122 (79.2)	105 (85.4)	227 (81.9)
It is beneficial to your health to drink milk every day. [yes]	126 (81.8)	98 (79.7)	224 (80.9)
It is beneficial to your health to eat more high-energy snacks. [no]	74 (48.1)	76 (61.8)	150 (54.2)

**Table 2:** Weight-control attitude of Chinese adolescents

Items	Number (%)		
	Male (*n* = 154)	Female (*n* = 123)	Total (*n* = 277)
To what extent do you think obesity is bad for health? [great or rather great]	79 (51.3)	62 (50.4)	141 (50.9)
Are you satisfied with your weight status? [no or rather no]	63 (40.9)	60 (48.8)	123 (44.4)
Do you want to change your present weight status? [yes or rather yes]	89 (57.8)	85 (69.1)	174 (62.8)
Do you believe you can achieve an ideal weight status through effort? [yes or rather yes]	79 (51.3)	71 (57.7)	150 (54.2)

**Table 3:** Median score for weight-control behaviours

Domains	Median (IQR)		Z stat^a^	*P*-value^a^
	Male (*n* = 154)	Female (*n* = 123)		
Dietary choices (DC)^b^	20.0 (8.3)	22.0 (10.0)	−0.92	0.359
Self-monitoring (SM)^c^	6.0 (9.0)	4.0 (7.0)	−2.45	**0.014**
Physical activity (PA)^d^	10.0 (9.0)	9.0 (7.0)	−2.11	**0.035**
Psychological coping (PC)^c^	11.0 (9.0)	10.0 (9.0)	−0.28	0.779

a Mann-Whitney U test, *P* < 0.05 indicates significance

b Range of scores for DC (0 to 40, the higher the score, the more frequent the behaviours)

c Range of scores for SM and PC (0 to 28, the higher the score, the more frequent the behaviours)

d Range of scores for PA (0 to 24, the higher the score, the more frequent the behaviours)

**Table 4:** Body-image perception for both males and females

Body-image perception	Male (*n* = 154)		Female (*n* = 123)		Total (*n* = 277)	
	*n*	%	*n*	%	*n*	%
Desirability to be bigger	45	29.2	10	8.1	55	19.9
Satisfied	48	31.2	46	37.4	94	33.9
Desirability to be smaller	61	39.6	67	54.5	128	46.2

**Table 5:** Current and ideal body-image perceptions

Stunkard’s Figure Rating Scale^a^	Median (IQR)		
	Male (*n* = 154)	Female (*n* = 123)	Total (*n* = 277)
Current body image	4 (3)	4 (1)	4 (2)
Ideal body image	4 (1)	3 (1)	4 (1)

a Range of scale: 0–9 (the bigger the scale, the bigger the body size)

**Table 6:** Comparison of weight-control behaviours between perceived current: ideal body-image groups

Perception	Median (IQR)			*X*^2^ stat (df)	*P*-value^a^
Weight-control behaviours	Bigger (*n* = 55)	Satisfied (*n* = 94)	Smaller (*n* = 128)		
Dietary choices (DC)	21.0 (7.0)	20.0 (10.5)	21.0 (8.8)	0.06 (2)	0.973
Self-monitoring (SM)	4.0 (8.0)	6.0 (10.5)	5.0 (6.8)	2.97 (2)	0.226
Physical activity (PA)	10.0 (11.0)	10.0 (9.0)	9.0 (7.0)	5.87 (2)	0.053
Psychological coping (PC)	9.0 (12.0)	11.0 (10.0)	11.5 (8.0)	1.71 (2)	0.425

a Kruskal-Wallis test, *P* < 0.05 indicates significance
